# Differential Effects of Dabigatran and Warfarin on Bone Volume and Structure in Rats with Normal Renal Function

**DOI:** 10.1371/journal.pone.0133847

**Published:** 2015-08-04

**Authors:** Maria Fusaro, Luca Dalle Carbonare, Adriana Dusso, Maria Vittoria Arcidiacono, Maria Teresa Valenti, Andrea Aghi, Sabina Pasho, Maurizio Gallieni

**Affiliations:** 1 National Research Council (CNR)–Neuroscience Institute, Padova, Italy; 2 Department of Medicine, Section of Internal Medicine D, University of Verona, Verona, Italy; 3 Division of Experimental Nephrology, IRB Lleida (Lleida Institute for Biomedical Research), Lleida, Spain; 4 Nephrology Unit, University of Padua, Padua, Italy; 5 Nephrology and Dialysis Unit, Ospedale San Carlo Borromeo and Department of Clinical and Biomedical Sciences "Luigi Sacco", University of Milano, Milano, Italy; 6 Department of Clinical and Biomedical Sciences "Luigi Sacco", University of Milano, Milano, Italy; Université de Lyon—Université Jean Monnet, FRANCE

## Abstract

**Background:**

Warfarin, a widely used anticoagulant, is a vitamin K antagonist impairing the activity of vitamin K-dependent Bone Gla Protein (BGP or Osteocalcin) and Matrix Gla Protein (MGP). Because dabigatran, a new anticoagulant, has no effect on vitamin K metabolism, the aim of this study was to compare the impact of warfarin and dabigatran administration on bone structure and vascular calcification.

**Methods:**

Rats with normal renal function received for 6 weeks warfarin, dabigatran or placebo. Bone was evaluated immuno-histochemically and hystomorphometrically after double labelling with declomycin and calcein. Aorta and iliac arteries were examined histologically.

**Results:**

Histomorphometric analysis of femur and vertebrae showed significantly decreased bone volume and increased trabecular separation in rats treated with warfarin. Vertebra analysis showed that the trabecular number was higher in dabigatran treated rats. Osteoblast activity and resorption parameters were similar among groups, except for maximum erosion depth, which was higher in warfarin treated rats, suggesting a higher osteoclastic activity. Therefore, warfarin treatment was also associated with higher bone formation rate/bone surface and activation frequency. Warfarin treatment may cause an increased bone turnover characterized by increased remodelling cycles, with stronger osteoclast activity compared to the other groups. There were no differences among experimental groups in calcium deposition either in aortic or iliac arteries.

**Conclusions:**

These findings suggest for the first time that dabigatran has a better bone safety profile than warfarin, as warfarin treatment affects bone by reducing trabecular size and structure, increasing turnover and reducing mineralization. These differences could potentially result in a lower incidence of fractures in dabigatran treated patients.

## Introduction

Warfarin is widely used to prevent venous thrombosis after orthopaedic surgery and strokes in non-valvular atrial fibrillation. Warfarin inhibits two important reactions of the vitamin K cycle at the quinone reductase and epoxide reductase levels, causing a functional shortage of vitamin K [1], a cofactor for γ-glutamyl carboxylase, the enzyme that activates several vitamin K-dependent proteins (VKDP) through γ-carboxylation, including Matrix Gla Protein (MGP), an inhibitor of vascular calcification [2, 3], as well as osteocalcin (BGP) and other osteoblast specific proteins, involved in proper bone mineralization during bone formation [4]. However, specific ablation of γ-glutamyl carboxylase in osteoblast has demonstrated that γ-carboxylation is not a pre-requisite for the bone protective effects of vitamin K [4]. Indeed, vitamin K benefits on bone health also include a positive calcium balance [5] and synergy with vitamin D bone forming actions [5–7]. Therefore, warfarin induced vitamin K deficiency increases the risk of developing osteoporosis and bone fragility [8].

Dabigatran is a direct inhibitor of thrombin (serine protease), blocking the conversion of fibrinogen into fibrin and thereby preventing thrombosis [9]. Unlike warfarin, dabigatran-driven thrombin inhibition does not interfere with the vitamin K cycle, thus preventing the risk of vascular calcifications, abnormal skeletal integrity and bone fractures caused by vitamin K deficiency.

The aim of this study was to compare the impact of warfarin and dabigatran on bone structure and on arterial calcifications in rats with normal renal function.

## Materials and Methods

### Animal Protocols

Experimental procedures were carried out following the guidelines for animal experiments established at the IRB Lleida and specifically approved by the Animal Study Committee at Lleida Institute, in agreement with the EU Directive 2010/63/EU for animal experiments.

Thirty-four female Sprague-Dawley (SD) rats, 10 weeks old, were randomly divided in three groups as follows: 1) *Normal Controls (Untreated)*: Rats (n = 10) were fed a control diet containing vitamin K3, at the concentration of 8 mg/kg. Assuming the average intake of chow for a rat is 15 to 30 g/day, vitamin K3 intake was 120 to 240 μg/day. The diet also contained 1000 IU/kg of vitamin D3, 1.05% calcium, 0.2% magnesium, and 0.8% phosphorus. 2) *Dabigatran Treatment*: Rats (n = 10) were fed the same control diet supplemented with dabigatran etexilate at a concentration of 1.0 mg/g of chow. 3) *Warfarin Treatment*: Rats (n = 14) were fed the control diet and received warfarin in the drinking water to reach a concentration sufficient to obtain an International Normalized Ratio (INR) between 2 and 3. A number equal to 14 animals was chosen for this group considering the possibility of death due to a high INR.

Specifically, upon arrival to the conventional animal facility, (12:12 hours dark:light cycle), rats, 2 to 3 in each cage, were subjected to a 4-day period of adaptation to the new control chow diet in order to obtain the average daily water intake to estimate the final concentration of warfarin in the drinking water required to achieve the desired INR starting from the reported warfarin supplementation of 0.6 mg/kg rat. Rats were treated as indicated for 6 weeks. INR tests in the warfarin-treated group were conducted in average every 3 days, in order to ensure proper adjustment of the oral dose of warfarin to avoid INR higher than 3. The warfarin dose was progressively reduced to 0.2 mg/kg, with an average administration of 0.255 ± 0.001 mg/kg. At the end of the study, rats were moved to the surgery room and anesthetized with the recommended dose of ketamine 10%: xylazine 2% cocktail intraperitoneally administered (ketamine 0.09g/kg of rat weight, xylazine 0.01g/kg of rat weight) to collect blood from the abdominal aorta. Then, euthanasia was performed by exsanguination under deep anesthesia. Control rats and dabigatran treated rats underwent Hemoclot tests in duplicate, to assess the anticoagulation activity of dabigatran and to quantify concentrations of dabigatran in plasma, as previously described [10]. Serum and plasma were kept at -20°C for further analysis.

### Vascular Calcification Studies

At necropsy, samples of aorta and iliac arteries were preserved for histological examination. Specifically, Von Kossa and Alizarin red staining were used to evaluate the degree of calcification with treatment.

### Bone Studies

Bone labelling was performed using declomycin and calcein, injected i.p. at a concentration of 15 mg/kg body weight, at 10 and 3 days prior to sacrifice, respectively.

Femur, tibia and vertebrae were collected and stored in ethanol for immuno-histochemical and morphometric analysis of bone remodelling.

### Bone Histomorphometry

**Fig 1:**
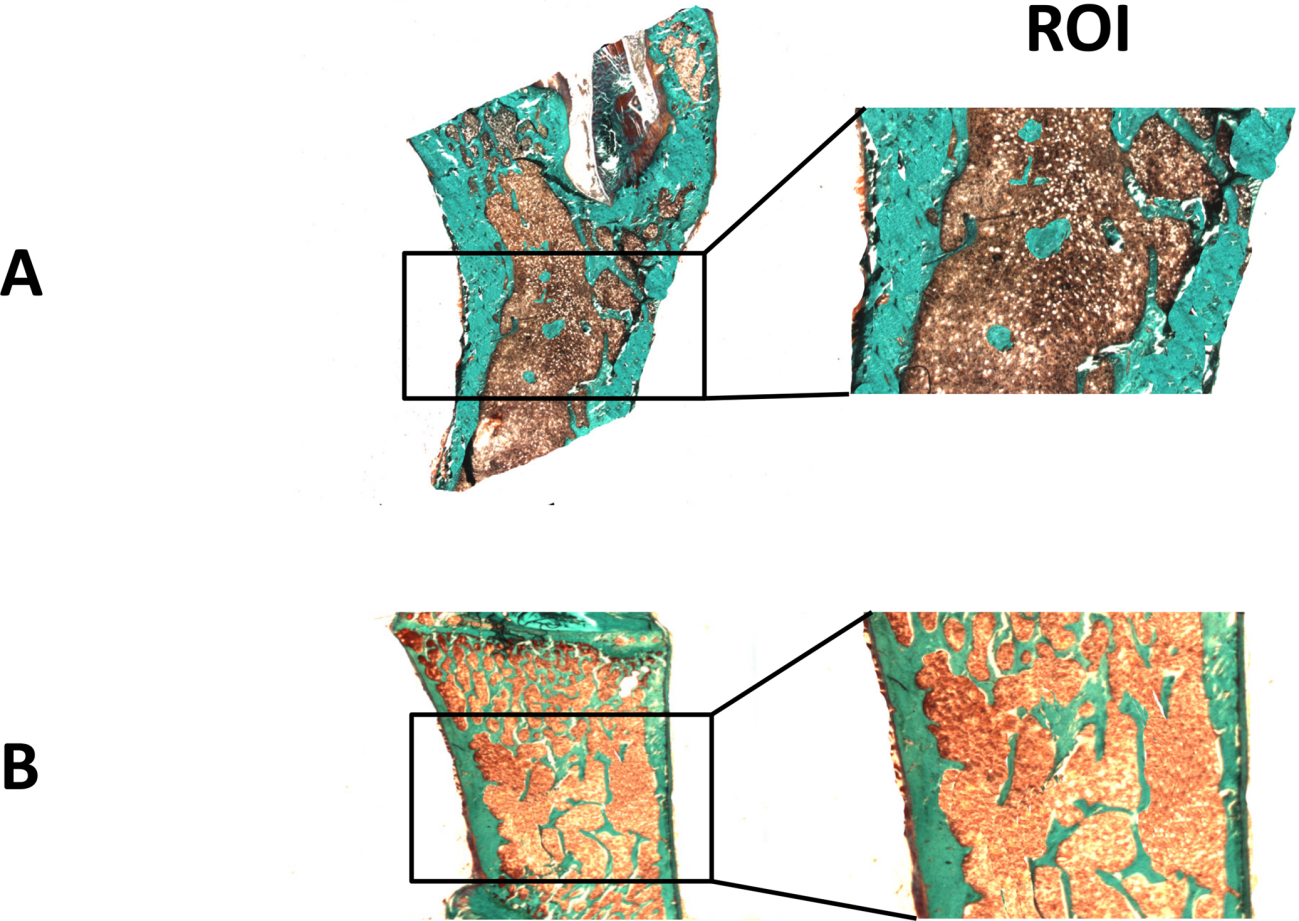
Regions of Interest (ROI) for histomorphometric analyses for femur (1A) and vertebra (1B). For femur analysis, we analyzed the trabecular and cortical bone (in green) of the secondary spongiosa area between 2 and 4 mm distal to the growth plate-metaphyseal junction in the upper part of the diaphysis. Concerning the vertebrae, we selected lumbar vertebrae and considered the middle area in frontal sections to evaluate trabecular and cortical bone parameters.

Rat femurs and vertebrae were embedded undecalcified in a methyl-methacrylate resin (Merck 800590, Germany). Bone sections were cut using a microtome (Leica RM2265, Leica Microsystems, Wetzlar, Germany) equipped with a carbide-tungsten blade, stained with Goldner’s stain, and mounted on microscope slides for histomorphometric measurements. The sections were obtained from three different levels of the methyl-methacrylate block, each separated by a thickness of 250 μm. Histomorphometric results were calculated as the mean of the values obtained from the three different levels as an approximation to a 3-D evaluation. This also avoids replicating the sampling of any single bone remodelling unit. For femur analysis, we analyzed the trabecular and cortical bone of the secondary spongiosa area between 2 and 4 mm distal to the growth plate-metaphyseal junction in the upper part of the diaphysis (Fig 1A), as in [11]. Concerning the vertebrae, we selected lumbar vertebrae and considered the middle area in frontal sections to evaluate trabecular and cortical bone parameters (Fig 1B).

Measurements were performed by means of an image analysis system consisting of an epifluorescent microscope (Leica DM 2005, Leica Microsystems, Wetzlar, Germany) connected to a digital camera (Leica DFC 420C, Leica Microsystems, Wetzlar, Germany) and a computer equipped with a specific software for histomorphometric analyses (Bone 3.5, Explora Nova, La Rochelle, France). Histomorphometric parameters were reported in accordance with the ASBMR Committee nomenclature [12].

Osteoid Surface (OS/BS, %), Osteoid Thickness (O.Th, μm), Osteoblast Surface (ObS/BS, %) were measured as static parameters of bone formation. Maximum Erosion Depth (MAXEDe, μm), Erosion Surface (ES/BS, %) and the number of osteoclasts (N.Oc/T.A /mm^2^) evaluated bone resorption. Bone Formation Rate (BFR/BS) and Activation Frequency (AcF) evaluated dynamic parameters of bone formation, reflecting bone turnover. Bone Formation Rate (BFR/BS) was calculated as Mineralizing Surfaces (MS/BS) X Mineral Apposition Rate (MAR), and expressed in μm^3^/μm^2^/day [12].

Mineral apposition rate (MAR) was calculated as the distance between at least 3 midpoints of two consecutive labels, divided by the time between the two tetracycline administrations. MS/BS was calculated as the double-labeled surface (dLS/BS) plus one half of the single-labeled surface (sLS/BS) expressed as a function of total bone surface.

Thickness results were adjusted for the obliquity of sections by multiplying by π/4. To assess trabecular microarchitecture, trabecular number (Tb.N), trabecular separation (Tb.Sp, μm), and trabecular thickness (Tb.Th, μm) were measured [13]. After skeletonization, the trabecular network was evaluated by measuring the connections between nodes (points at which three or more trabeculae join), connection branches (struts), terminal or free-ends branches (termini) and by calculating number of nodes (N.Nd/TV), and nodes/termini ratio (Nd/Tm), as previously described [14]. Indirect parameters of microarchitecture were also assessed including: Marrow Star Volume (MSV, N/mm^3^), which is the mean volume of all the parts of an object that can be unobscured in all the directions from a point inside the object [15]; Fractal Dimension (D), which describes how an object fills a space according to its structure and which also evaluates bone structural anisotropy [16].

After thresholding the section to distinguish bone from marrow, the endosteal surface of the cortex was identified subjectively to exclude trabecular bone and include only bone with a typical cortical osteonal structure. Cortical porosity was calculated as a percentage of the total cortical area.

### Statistical Analysis

Results are expressed as mean ± SD or mean ± SEM. We performed Kolmogorov-Smirnov test and Levine’s test to verify the normal distribution of the results and the homogeneity of the variance, respectively. Based on the results of these tests, we used parametric test to analyze differences among groups by one-way analysis of variance (ANOVA). A p<0.05 was considered statistically significant. Differences among pairs of groups were tested with Bonferroni as a post-hoc test. Statistical analyses were performed by using SPSS for Windows version 21.0 (SPSS Inc., Chicago, IL, USA).

## Results

### Efficacy of Anticoagulant Treatment

Significant increases of INR and prothrombin time in the warfarin group vs. either dabigatran or controls were observed (Table 1).

**Table 1:** Anticoagulation parameters (S1 Dataset).

Treatment	Prothrombin Time (sec)	INR	Mean INR	Hemoclot test (sec)
Timing	End of study	End of study	Throughout study	End of study
**Control (n = 10)**	**9.6 ± 0.1**	**0.83 ± 0.01**	**n.a.**	**31.4 ± 0.4**
**Warfarin (n = 7)**	**43.0 ± 8.3** *	**4.31 ± 6.27** *	**2.8 ± 0.29**	**n.a.**
**Dabigatran(n = 10)**	**11.7 ± 0.4**	**1.05 ± 0.03**	**n.a.**	**48.9±2.0** #

Data are expressed as mean ± SEM

n.a. = not available.

* indicates p<0.001 vs. dabigatran or control

# indicates p<0.001 vs. controls

The starting warfarin dose of 0.6 mg/kg was excessive, resulting in elevated INR values and three deaths. Due to the short exposure to warfarin (4 days), these three animals were excluded from further analysis. Individualized dose reductions (average = 0.255) allowed obtaining the desired target INR between 2 and 3, with an overall mean INR of 2.8 ± 0.29. Warfarin treated rats had a mortality rate of 43% (in total, 6/14 rats died) with evidence of intra-hepatic bleeding at necropsy. No deaths occurred either in the dabigatran or the control group.

Results of the hemoclot tests at the end of treatment (Table 1) demonstrated effective anticoagulation achieved in the dabigatran treated rats. Results of the hemoclot (dTT)-assay indicated a dabigatran plasma concentration of 317.3±30.1 nmol/l (equivalent to 149.6±14.2 ng/ml).

### Vascular Calcifications

Under our experimental conditions, there was no evidence of arterial calcium deposition either in the aorta or in the iliac arteries among the three groups of rats.

### Bone Histomorphometry

Ten rats treated with dabigatran and ten control rats were available for histomorphometric analysis, while in warfarin treated group the analysis was performed on seven surviving rats.

#### Analysis of proximal femur

**Fig 2:**
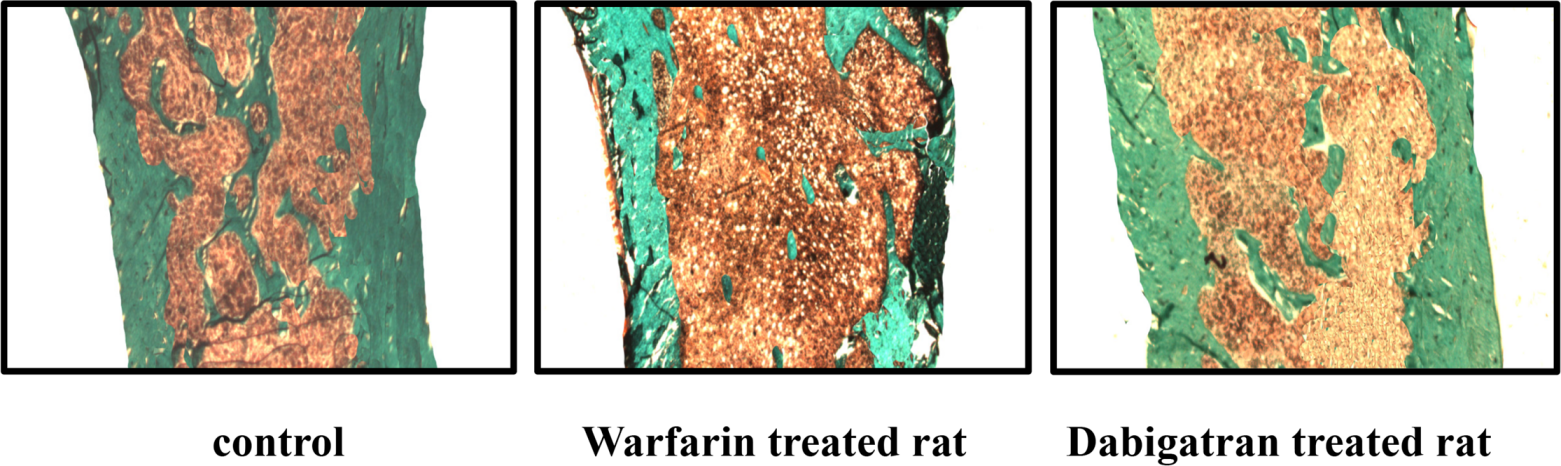
Dabigatran preserves femur volume and structure. Note the higher bone volume and trabecular thickness with lesser trabecular separation in rats treated with dabigatran compared to rats treated with warfarin. Bone of a representative normal control rat is also shown. (magnification 100X).

**Fig 3:**
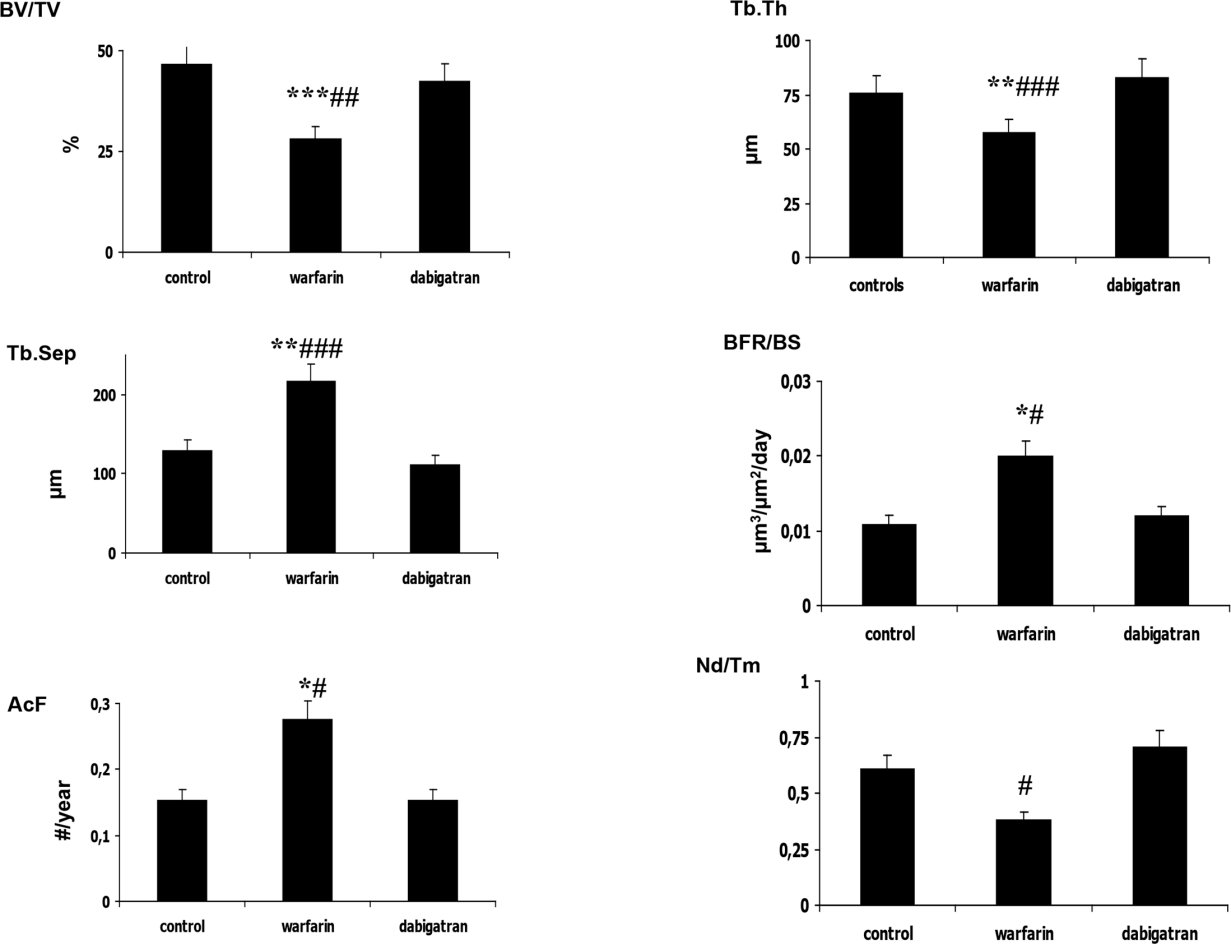
Significant histomorphometric results of structural and dynamic parameters in femur rats. In warfarin treated rats we observed lower bone volume and trabecular thickness with increased trabecular separation compared to dabigatran treated and control rats. In addition, treatment with warfarin was associated with a significant increase of turnover parameters (i.e. BFR/BS) and, in particular, activation frequency was higher in warfarin treated rats versus dabigatran treated or control rats. Microarchitecture, cortical thickness and porosity were similar among groups. Only rats treated with warfarin showed an alteration of bone volume and structure, suggesting increased bone fragility compared to dabigatran and control rats pattern. For the meaning of abbreviations see **Table 2**. *p<0.05, **p< 0.01, ***p<0.001 vs control; # p<0.05, ##p< 0.01, ### p<0.001 vs dabigatran.

**Fig 4:**
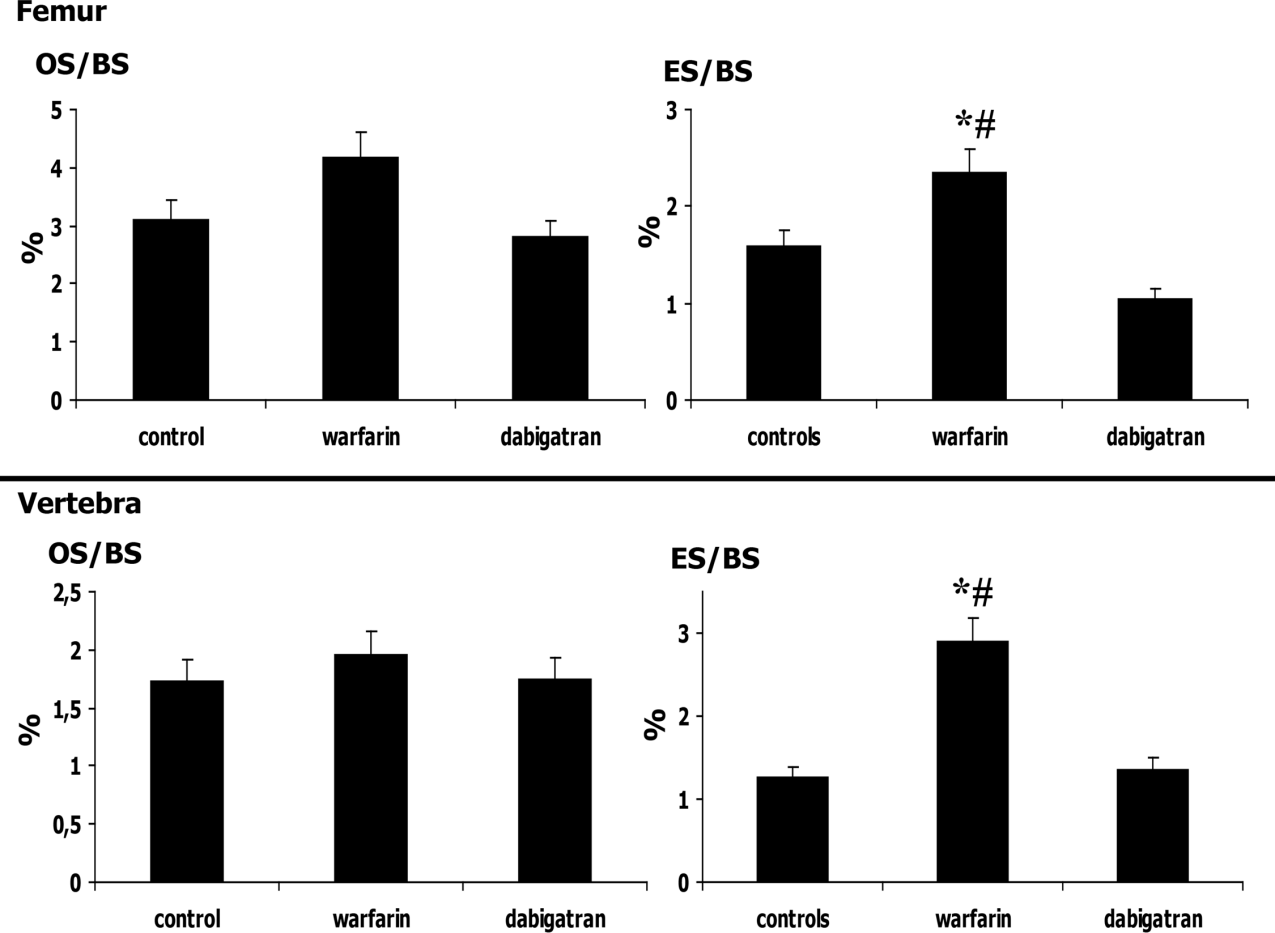
Significant histomorphometric results of cellular parameters in femur (upper panel) and vertebra (lower panel) analysis of rats. The main alteration of cellular parameters in warfarin treated rats was the increased osteoclast activity (ES/BS), although in femur a decreased osteoblast activity (OS/BS) was also detected. ES/BS: Erosion Surface / Bone Surface; OS/BS: Osteoid Surface / Bone Surface. *p< 0.01 vs control; #p<0.001 vs dabigatran; ##p< 0.005 vs dabigatran.

Parameters of structure showed decreased bone volume and increased trabecular separation in rats treated with warfarin with respect to dabigatran treated and control rats (Figs 2 and 3). Reduced osteoblast activity was detected in warfarin treated group (Fig 4). In addition, treatment with warfarin was associated with increased turnover, characterized by increased resorption parameters and higher BFR/BS as a result of a similar MAR with increased MS/BS. Consistently with these results, activation frequency (AcF) was also higher in warfarin treated group than in dabigatran treated and control rats (Fig 3).

There were no substantial differences in microarchitecture among groups (Fig 2), with similar node number/tissue volume, marrow star volume, and fractal dimension and a little difference in node/termini ratio (N.Nd/TV, MSV, D and Nd/Tm D, Table 2).

**Table 2:** Histomorphometric parameters in femur and vertebra (S2 Dataset and S3 Dataset).

*Femur*	Control (10)	Warfarin (7)	Dabigatran (10)
Trabecular Number (N)	**4.74±1.36**	**3.83±1.13**	**4.43±1.46**
Osteoid Thickness (O.Th., μm)	**8,39±2,23**	**9.39±1,47**	**8,73±1,83**
Osteoblast Surface/Bone Surface (Ob.S/BS, %)	**0,74±0,31**	**0,87±0,28**	**0,76±0,34**
Number of Osteoclast/Tissue Area (N.Oc/TA, /mm^2^)	**2.08±1.36**	**1.08±0.21**	**1.72±0.60**
Mineral Apposition Rate (μm/day)	**0.40±0.13**	**0.39±0.10**	**0.39±0.14**
Single Labelled Surface/Bone Surface sLS/BS (%)	**2,45±1,09**	**4,12±1,13** *	**2,72±1,07**
Double Labelled Surface/Bone Surface dLS/BS (%)	**1,85±1,07**	**3,52±1,15** *	**2,11±1,12**
MS/BS (%)	**3,18±1,47**	**5,58±1,69** *	**3,48±1,60**
Node Number/Tissue Volume (Nd.N/TV, %)	**10.75±6.30**	**5.79±2.00**	**8.40±6.04**
Marrow Star Volume (MSV, /mm^3^)	**0.06±0.07**	**0.07±0.09**	**0.02±0.03**
Fractal Dimension (D, #)	**1.49±0.10**	**1.54±0.16**	**1.58±0.09**
***Vertebra***			
Osteoid Thickness (O.Th, μm)	**8.55±2.21**	**10.98±1.08**	**10.85±2.56**
Osteoblast Surface/Bone Surface (Ob.S/BS, %)	**0.73±0.48**	**0.77±0.29**	**0.79±0.29**
Number of Osteoclast /Tissue Area (N.Oc/TA, /mm^2^)	**1.03±0.48**	**1.55±0.19**	**1.21±0.52**
Mineral Apposition Rate (μm/day)	**0.76±0.09**	**0.81±0.07**	**0.85±0.17**
Single Labelled Surface/Bone Surface sLS/BS (%)	**1,24±0,23**	**2,44±0,41** **	**1,27±0,50**
Double Labelled Surface/Bone Surface dLS/BS (%)	**0,64±0,33**	**1,84±0,44** **	**0,67±0,61**
MS/BS (%)	**1,25±0,34**	**3,01±0,61** **	**1,30±0,76**
Node Number/Tissue Volume (Nd.N./TV %)	**14.96±12.44**	**9.70±5.11**	**19.51±13.86**
Node/Termini ratio (%)	**0.52±0.15**	**0.59±0.27**	**0.78±0.34**
Marrow Star Volume (MSV/mm^3^)	**0.01±0.01**	**0.05±0.08**	**0.01±0.03**
Fractal Dimension (D, #)	**1.56±0.07**	**1.60±0.07**	**1.56±0.06**

*p< 0.05 vs control and dabigatran groups;

** **p<0.001 vs control and dabigatran groups.**

#### Analysis of Vertebrae

**Fig 5:**
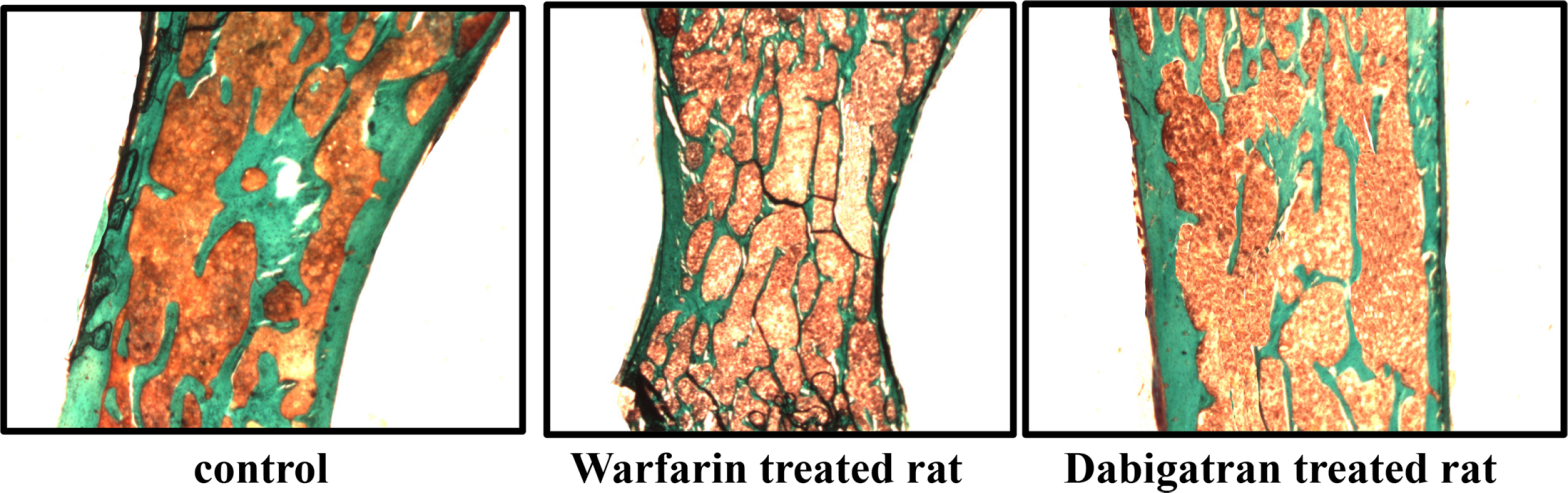
Dabigatran preserves vertebral volume and structure. According to what observed in the femur, analysis of vertebrae showed increased bone volume, trabecular thickness and number, with lesser trabecular separation in rats treated with dabigatran with respect to warfarin treated study animals. Bone of a representative normal control rat is also shown. No significant differences in microarchitecture were observed between study groups. Warfarin treated rats showed thinner trabeculae with increased separation in the marrow space, highlighting a potentially increased fragility, despite similar microarchitecture. (Magnification 100X).

**Fig 6:**
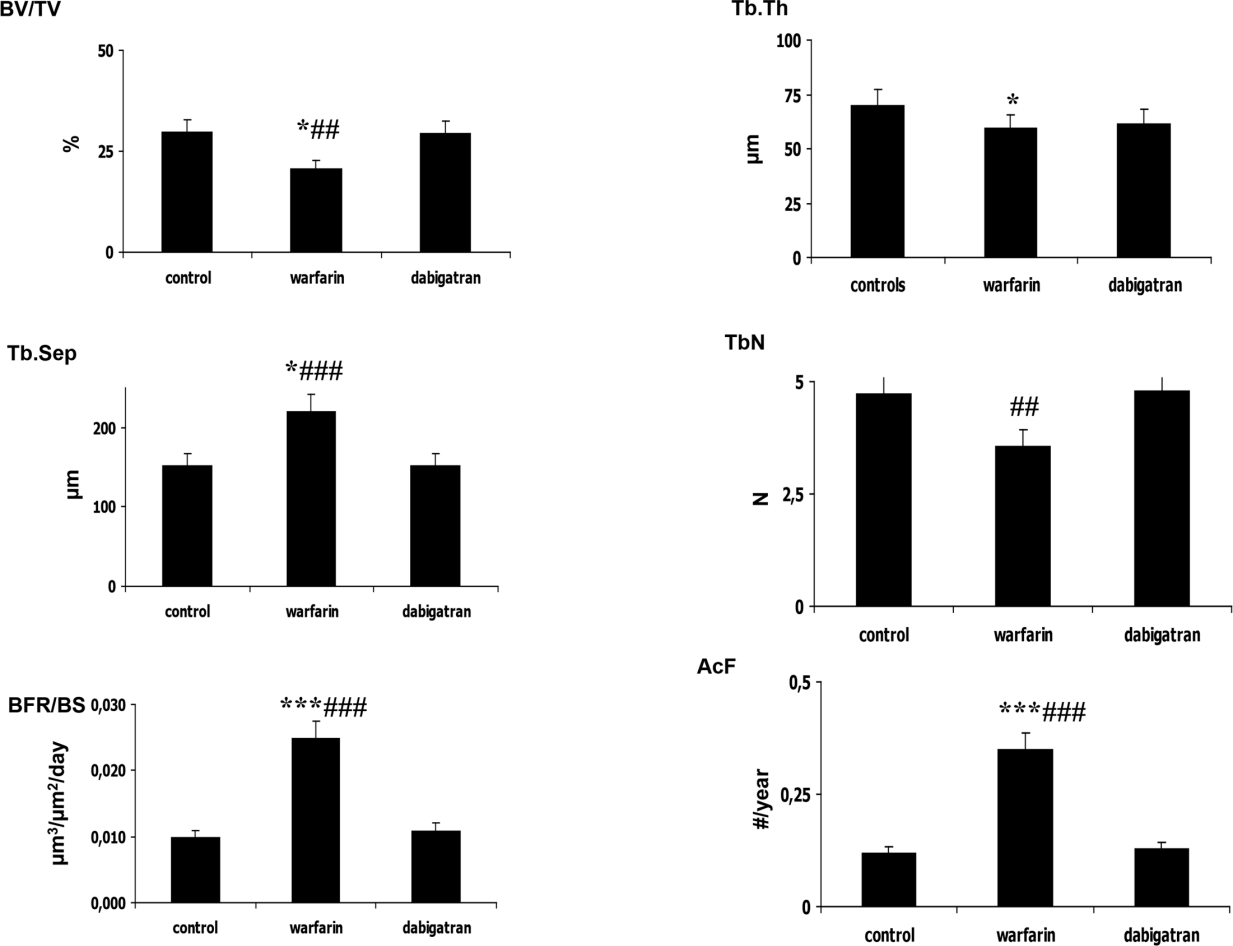
Significant histomorphometric results of structural and dynamic parameters in vertebrae of rats. Similar to the findings in femur, in warfarin treated rats we observed lower bone volume and trabecular thickness with increased trabecular separation compared to dabigatran treated and control rats. In addition, treatment with warfarin was associated with a significant increase of turnover parameters, i.e. BFR/BS and activation frequency. Microarchitecture, cortical thickness and porosity were similar among groups. Only rats treated with warfarin showed an alteration of bone volume and structure, suggesting increased bone fragility compared to dabigatran and control rats pattern. *p<0.05,** p< 0.01, ***p<0.001 vs control; #p<0.05, ## p< 0.01, ###p<0.001 vs dabigatran.

The analysis of bone structure showed significant decreases in bone volume and increases in trabecular separation in rats treated with warfarin compared to dabigatran treated and control rats (Fig 5). In addition, trabecular number was lower in the warfarin treated group than in the dabigatran group. Concerning the remodelling, we observed significant difference in osteoblast activity (BFR/BS, Fig 6) and resorption parameters (ES/BS, Fig 4) associated to higher Ac.F in warfarin-treated groups vs both others, suggesting that warfarin may cause an increased turnover characterized by increased remodelling cycles, with increased osteoclast activity with respect to other groups. No substantial differences in microarchitecture, (N.Nd/TV, Nd/Tm, MSV, and D) were observed among groups, even though there was a tendency towards altered microarchitecture parameters with warfarin treatment. Thus, in the vertebrae, treatment with warfarin decreased bone volume with increased trabecular separation as a result of increased turnover and osteoclast activity, while treatment with dabigatran maintained the parameters of structure and turnover similar to those in the control group, showing, as a result, a bone sparing effect versus warfarin.

No differences in cortical thickness and porosity were found among groups, indirectly suggesting that neither warfarin nor dabigatran affected parathyroid activity (data not shown).

## Discussion

Warfarin, a vitamin K antagonist (VKA), reduces the risk of stroke in patients with atrial fibrillation but has some limitations, including an increased risk of bleeding and the need for close monitoring. Such limitations led to the development of new oral anticoagulants. Dabigatran is an oral direct thrombin inhibitor, whose administration to patients with atrial fibrillation was associated with rates of stroke and systemic embolism similar as warfarin, but with lower rates of major haemorrhage [17]. Warfarin as a VKA interferes with vitamin K actions, including the carboxylation of vitamin K dependent proteins MGP and BGP causing critical adverse side effects on bone and vascular health [18–20]. Dabigatran and other new oral anticoagulants do not affect vitamin K metabolism. Thus, besides reducing major haemorrhage, they might also attenuate serious adverse side effects induced by vitamin K deficiency, as vascular calcifications and bone fractures.

This experimental study in rats shows that treatment with warfarin is associated with significant bone abnormalities, differently from dabigatran and placebo treatment, but is not associated with vascular calcification. The unexpected findings of absent calcification are in accordance with a recent study by McCabe et al. demonstrating warfarin-induced vascular calcification in CKD rats but not in animals with normal renal function [21] maybe because doses of warfarin were lower than those previously employed to study the calcifying effects of anticoagulation treatment. Warfarin at high doses causes rapid calcification of rat arteries, with aortic calcification seen after only 2 weeks of warfarin treatment [3]. In DBA/2 mice [22] and in apolipoprotein E–deficient non-CKD mice [23], a recognized model of atherosclerosis, vascular calcifications developed in less than 4 weeks with the administration of supra-therapeutic doses of warfarin (3.0 mg/g of diet) even when the diet was supplemented with vitamin K1 (1.5 mg/g of diet). Such generation of vascular calcifications in response to warfarin administration in mice is restricted to a genetic background, because the same protocol applied on C57BL/6 mice did not result in comparable generation of vascular calcifications [22]. In both studies [22,23], vitamin K1 was co-administered to avoid internal bleeding due to the high warfarin dosage, because vitamin K1 antagonizes warfarin preferentially in liver but not in extra-hepatic tissues [24]. Our approach was different, as we reproduced the usual clinical setting, which is adapting warfarin dosage to keep the prothrombin INR in the 2 to 3 range. Furthermore, while high warfarin doses may block peripheral vitamin K activity, thereby inhibiting the γ-carboxylation of vascular MGP and of bone osteocalcin, the endogenous conversion of vitamin K1 to menaquinone-4 may contribute to the direct, osteocalcin-independent protective effects on bone [25,26].

Although a more physiological approach was used in our study, standard animal diets contain some vitamin K3 (menadione), which may have contributed to maintain synthesis of carboxylated MGP. Vitamin K1 (phylloquinone) and vitamin K2 (menaquinones) are two naturally occurring forms of vitamin K, while vitamin K3 (menadione) is an intermediate endogenous metabolite of vitamin K, that also exists as a synthetic compound.

Vitamin K1 is the main (>90%) dietary source of vitamin K, but its tissue concentrations are remarkably low compared with those of vitamin K2, the major form (>90%) of vitamin K in tissues. Vitamin K1 appears to be converted into vitamin K2 in extra-hepatic tissues. In this process, vitamin K3 is a catabolic product of vitamin K1 and a major source of tissue vitamin K2 [27–30]. Even in humans, vitamin K1 can be endogenously converted to vitamin K2, either directly or through vitamin K3. Nakagawa et al. identified the human enzyme responsible for menaquinone-4 biosynthesis, UbiA prenyltransferase containing 1 (UBIAD1), a human homologue of Escherichia coli prenyltransferase menA [31]. In mice, this enzyme was also localized in endoplasmic reticulum and ubiquitously expressed in several tissues. Interestingly, experimental menaquinone-4 biosynthesis by UBIAD1 was not affected by warfarin [31]. The absence of calcifications in our experimental setting raises two important considerations for therapy. First, that previous models of warfarin-induced vascular calcifications may not accurately reflect the clinical setting, where lower doses of warfarin are used and a longer period of vitamin K antagonism and abnormal bone metabolism may be necessary to detect an increased arterial calcium deposition. Second, bone metabolism appears to be more sensitive than the vasculature to the vitamin K inhibitory activity of warfarin.

Previous studies have established that vitamin K deficiency significantly affects bone health [26, 32–36]. While there is some controversy as to whether warfarin decreases bone mineral density [33], there is agreement on warfarin-induced risk of rib and vertebral fractures in elderly patients [34–36], possibly by increasing skeletal fragility through the onset of vitamin K deficiency. Thus, the increased risk of fractures in warfarin-treated patients might depend on the impairment of bone quality rather than quantity [26, 37], and on the duration of treatment, because there was no increased risk of osteoporotic fractures in patients prescribed warfarin for less than a year [36]. Interestingly, different from vertebral fractures, long-term therapy with warfarin did not increase the risk of hip fractures in the elderly [38, 39]. In an experimental setting, Sugiyama et al. [26] partially explained this contradictory finding by considering the fundamental role of physical exercise and mechanical loading, suggesting that long-term warfarin therapy weakens rib and vertebrae by impairing cortical bone quality. In addition, higher mechanical stimuli in the hip prevent loss of bone strength through a compensatory adaptation of cortical bone structure. However, in view of the direct impact of vitamin K on skeletal integrity, a direct role of abnormal vitamin K metabolism cannot be ruled out.

Our detailed analysis of the negative effects of warfarin treatment compared to dabigatran on bone structure and quality expands the current understanding of changes in bone histomorphometry during warfarin treatment. Amizuka [40] reported an inhibiting effect of warfarin on mineralization and lower osteocalcin content in bone, with defective mineralization of the osteoid. Haffa et al. evaluated markers of bone turnover, density, and strength in rats with warfarin induced vitamin K insufficiency and reduced deposits of osteocalcin in bone, without hindering the attainment of peak bone mass [41].

The role of osteocalcin in bone remodeling and quality is still debated. In an experimental study on the function of osteocalcin in bone, Lian et al. demonstrated that osteocalcin-deficient bone particles obtained from warfarin treated rats were resistant to resorption when implanted subcutaneously in normal rats [42], suggesting that osteocalcin is an essential component for bone matrix to elicit progenitor-cell recruitment and the differentiation necessary for bone turnover. However, Serre et al. could not reproduce their findings [43], and Karsenty´s group reported an increased rather than reduced bone mass in the osteocalcin null mice [44].

In an experimental setting in monkeys on long-term warfarin anticoagulation, Binkley et al did not find modifications of serum markers of bone turnover or BMD measured by DEXA [45]. Again, these finding point to a qualitative, rather than quantitative bone damage of vitamin K inhibition. Accordingly, Cheung et al. in a randomized controlled trial of vitamin K1 supplementation (5 mg/day) in postmenopausal women found no differences in BMD values between the placebo and vitamin K groups, but fewer women in the vitamin K group had clinical fractures [46].

A limitation of this study is the lack of bone biochemical markers. However, histological analysis is a more powerful tool in detecting subclinical effects on bone of the tested drugs.

Results from our bone histomorphometry studies indicate that warfarin augments bone turnover through an increase of osteoclastic activity (as indicated by the finding of deeper lacunae) rather than by an increase in the number of osteoclasts. Bone microarchitecture was not affected in our experiments. However, after a longer exposure to warfarin, which is associated with increased osteoclastic activity, significant decreases in bone volume and increases in trabecular separation might determine alterations in bone quality and a higher propensity to fractures.

On the other hand, the lack of an increased osteoclast activity along with the preservation of structural parameters in the dabigatran treated group support a different impact of the two treatments on bone, with dabigatran being safer in this context.

## Conclusions

Treatment with warfarin in rats was associated with decreased bone volume, increased trabecular separation and higher turnover compared to that observed in dabigatran-treated or control rats. These findings suggest that dabigatran has a better bone safety profile than warfarin. Since warfarin treatment affects bone by reducing trabecular size and structure, increasing turnover and reducing mineralization, these differences could translate into a lower incidence of fractures in dabigatran treated patients.

## Supporting Information

S1 Dataset Coagulation Dataset. (XLSX)

S2 Dataset Dataset Femur. (XLS)

S3 Dataset Dataset Vertebra. (XLS)
